# Risks of accidents at work in health professionals

**DOI:** 10.1186/1753-6561-5-S6-P287

**Published:** 2011-06-29

**Authors:** J Da Silva, CN Shimura, A Giordani, NEPECISS

**Affiliations:** 1 Escola de Enfermagem de Ribeirão Preto - USP, Ribeirão Preto, Brazil

## Introduction / objectives

Work accidents are events that occur in the environment of workers according to their activities. The work is an important human activity which brings a gain both professional and personal, but submittingfails as well . This study aims to analyze the main occupational risks that health professionals are subjected in their work activities, and reflect measures that can prevent these accidents at work.

## Methods

A method integrative review of the literature through the selection of articles in databases such as Medline, CINAHL and Lilacs, using as key words "accident", "work" and "health", totaling 12 publications in the last ten years.

## Results

The result was obtained several aspects related to workplace accidents, among them: there are general estimates that each year approximately two million women and men die as a result of occupational accidents and work-related diseases. Health professionals are exposed to a number of risks, such as chemical, physical, biological, psychosocial, ergonomic, mechanical and actual accidents. According to the scholars of the topic in question biohazards are the main generators of health and risk premiums for these workers, because once in contact with bodily fluids from customers such as blood, there may be transmission of pathogenic microorganisms, and the virus HIV, hepatitis B and C, the main of them.

## Conclusion

It is important then, to emphasize that preventive measures should be adopted in preventing contamination through accidents, such as vaccination, use of personal protective equipment (PPE), tools for professionals and compliance with standard precautions. It is therefore crucial health professionals pay attention to as possible causes of accidents in their workplaces and, through simple measures such as use of PPE can significantly reduce these risks.

## Disclosure of interest

None declared.

